# Photosynthetic performance of symbiont-bearing foraminifera *Heterostegina depressa* affected by sunscreens

**DOI:** 10.1038/s41598-022-06735-1

**Published:** 2022-02-17

**Authors:** Michael Lintner, Michael Schagerl, Bianca Lintner, Matthias Nagy, Petra Heinz

**Affiliations:** 1 Department of Paleontology, University of Vienna, Vienna, Austria; 2 Department of Limnology and Oceanography, University of Vienna, Vienna, Austria

**Keywords:** Ecosystem ecology, Environmental sciences, Environmental chemistry

## Abstract

Foraminifera are abundant unicellular organisms that play an important role in marine element cycles. A large benthic foraminifer obligatory bearing photosymbionts is *Heterostegina depressa*. We studied potential impacts of sunscreens available on the market on the activity of photosymbionts on *H. depressa* by means of pulse-amplitude modulated (PAM) fluorescence microscopy. We included four different sunscreens, with two of them sold as “conventional” and two more stated as “eco-friendly”. Further, the impact of pure Ensulizole (phenylbenzimidazole sulfonic acid) was tested, which is a common agent of sunscreens. Foraminifera were incubated at varying concentrations (10, 50 and 200 mgL^−1^) of different sunscreens and the pure Ensulizole for 14 days. The photosynthetic performance was measured after 1,3, 7 and 14 days. Pure Ensulizole had a strong negative impact on the photobionts, which was reflected by a significant reduction of the areal fluorescence signal. “Eco-friendly” sunscreens affected the health of foraminifera more severely compared to “conventional” ones. We assume that metal nanoparticles like titanium dioxide or zinc oxide of “eco-friendly” sunscreens are causing this impact, because these substances were already classified as toxic for several microorganisms.

## Introduction

Foraminifera are unicellular shelled microorganisms and abundant in the benthic and pelagic zone of oceans^1,2^. Only 10% of all known foraminifera families are able to host endosymbionts, but actually those are most important for carbonate production (Lee and Anderson 1991). Tropical larger foraminifera which occur in coral reefs worldwide (e.g.,^3^) and planktonic foraminifera are both able to host endosymbiont and made up 20.5% of the estimated carbon production of the year (Langer 1997). The advantage for foraminifera to host symbionts is mathematically stated by Hallock (1981) and describes that the algal symbionts provide substances like sugars and glycerol for the host (Falkowski et al. 1993). Large benthic foraminifera (LBF) are negatively affected by varying environmental parameters like temperature or pH, which further has a negative impact on the carbonate equilibrium in coral reefs^4^. Due to the fact, that symbiont bearing foraminifera are able to secrete high-magnesium calcite for their tests, they can be used as “first responders” in reef communities to define a decreasing saturation state of seawater relative to ocean acidification^4–6^.

Nutrient uptake of foraminifera depends on several factors like the size of food particles^7^, water temperature^8^ and salinity^9^. It turned out that sea surface temperature and salinity are key environmental factors that regulate the distribution of large benthic foraminifera, which might lead to a shift in distribution patterns due to climate change^10^, Occhipinti-Amborgi^11^. Obviously, one of the most important environmental parameters for photosymbiont bearing foraminifera is the light supply^12^. The depth distribution of LBFs depends on seasonal variations in the water transparency^13^. Some families of LBFs (Amphisteginidae and Nummulitidae) can even be found in 130 m water depth with lower than 1% of the surface photosynthetically active radiation^14^. All these aspects highlight the importance of investigations on LBFs and will help to understand the relation between reef systems and global change.

This study focuses on the species *Heterostegina depressa*, which is known as alien species in the Mediterranean Sea^15^. *Heterostegina depressa* is currently found in the western part of the Mediterranean Sea^15^, and it is important to investigate it, as this species is actually spreading into new regions eastwards. Compared to other foraminifera of the Mediterranean Sea, this large symbiont-bearing and calcifying taxon is better adapted to increasing temperatures (Schmidt et al. 2016). *Heterostegina depressa* hosts obligatory photosynthetic symbionts (diatoms), which are essential for their foraminiferal metabolic activity. The photosynthetic performance of these photobionts is largely influenced by physical and chemical parameters. We studied potential impacts of man-made sunscreens, which might be found at higher concentrations especially in beach areas and river deltas. McCoshum et al.^16^ proved that sunscreens have negative effects on algal communities in natural waters. The estimated worldwide production of UV filters amounts up to 10 000 metric tons per year and has the potential of getting disposed in natural environments^17^. Some agents such as the common organic sunscreen Ensulizole (phenyl benzimidazole sulfonic acid) are resistant against microbial decomposition processes^18^. Ensulizole is used as pure UVB filter in cosmetics^19^. It is water-soluble and feels lighter on skin than non-water-soluble components. Therefore, it is widely used in products where a non-greasy finish is the aesthetic goal. Studies have shown that Ensulizole can have negative effects on organisms, for example by damaging DNA through the generation of reactive oxygen species^20^. As these substances are toxic for organisms including the photosymbionts of foraminifera, their application may lead to reduced vitality of the foraminiferal host and finally outcompeting by other taxa. The current study intends to investigate potential impacts of sunscreen on the photosynthetic performance of foraminifera by means of the non-invasive technique of pulse amplified modulated fluorescence. We considered different sunscreens (both non-organic, conventional ones and eco-friendly sunscreens) and pure Ensulizole.

## Methods and work plan

Experiments were performed in six-well-plates. In each well, a single individuum was placed. For the preparation of the stock solution, 1 g of the respective sunscreen (Table 1) was added to 100 ml sterile filtered sea water and mixed on a magnetic stirrer for 24 h. From the stock solution, dilutions of 1 + 99 (100-fold, labelled as 100), 1 + 19 (20-fold, labelled as 20) and 1 + 4 (fivefold, labelled as 5) were prepared. A standard curve with clean Ensulizole in sterile seawater was prepared and the fluorescence (350 nm emission, 300 nm excitation) was measured using a spectrofluorometer (Shimadzu RF-5301PC) to define the concentration of Ensulizole in the samples. The calibration curve is c [µg] = 0.09962*[Intensity] − 0.21132 and shows an excellent correlation of r^2^ = 0.99998. Based on that, the Ensulizole concentrations of the sunscreens are described in Table 2. In addition, pure Ensulizole was analysed with concentrations of 10, 50, and 200 mg L^−1^, respectively. A set with only sterile seawater provided was run as a control. For each approach, six replicates were taken.

**Table 1:** Sunscreens used.

Component	Product name	Type
Sunscreen A	Nivea sun	Conventional
Sunscreen B	Nivea sun kids	Conventional
Sunscreen D	Eco cosmetics	Eco-friendly
Sunscreen F	Terra Naturi	Eco-friendly
Ensulizole E	CAS 27503-81-7	–
control C (no other additional component)	–	–

**Table 2:** Ensulizole concentrations of the tested sunscreens (standard deviations are given in parenthesis, n = 3).

Sunscreen	Ensulizole concentration [µg/L]	Sunscreen	Ensulizole concentration [µg/L]
A10	175.25 (0.05)	D10	0
A50	863.53 (0.09)	D50	0
A200	4088.69 (0.21)	D200	0
B10	118.98 (0.06)	F10	0
B50	580.66 (0.11)	F50	0
B200	2568.63 (0.30)	F200	0
E10	9220.24 (0.18)		
E50	52,293.00 (2.15)		
E200	276,179.25 (26.88)		

Foraminifera were incubated at a constant temperature (25 °C) and a light: dark regime of = 16 h: 8 h for three weeks. Overall photosynthetic performance of photosymbionts was done by means of variable chlorophyll fluorescence imaging of photosystem II (PSII; Imaging PAM Microscopy Version–Walz GmbH; excitation at 625 nm). The maximum quantum yield (Fv/Fm) is a ratio which describes the difference between maximum fluorescence and minimum fluorescence and will give information about the efficiency of the photosystem II^21^. This correlation is based on several biophysical models (e.g.,^22,23^) and can be used as a proxy for the activity of photosynthetic organisms^21^. In addition to maximum dark fluorescence yield (Fv/Fm), the photosynthetic area in each specimen was calculated. PAM-images were evaluated with the software WinControl-3 (Walz GmbH) and the change of photosynthetically active areas was measured using Image J (version 1.53 k,Java).

Specimens were measured at the start of the experiments (day 0) and after 1, 3 and 7 days in order to consider time-dependent changes of photobiont activity. For statistical analysis the software Statgraphics 18 was used to perform two-way ANOVA dealing with type of sunscreen and time (level of significance of 95%).

## Results

### The conventional sunscreens

**Figure 1:**
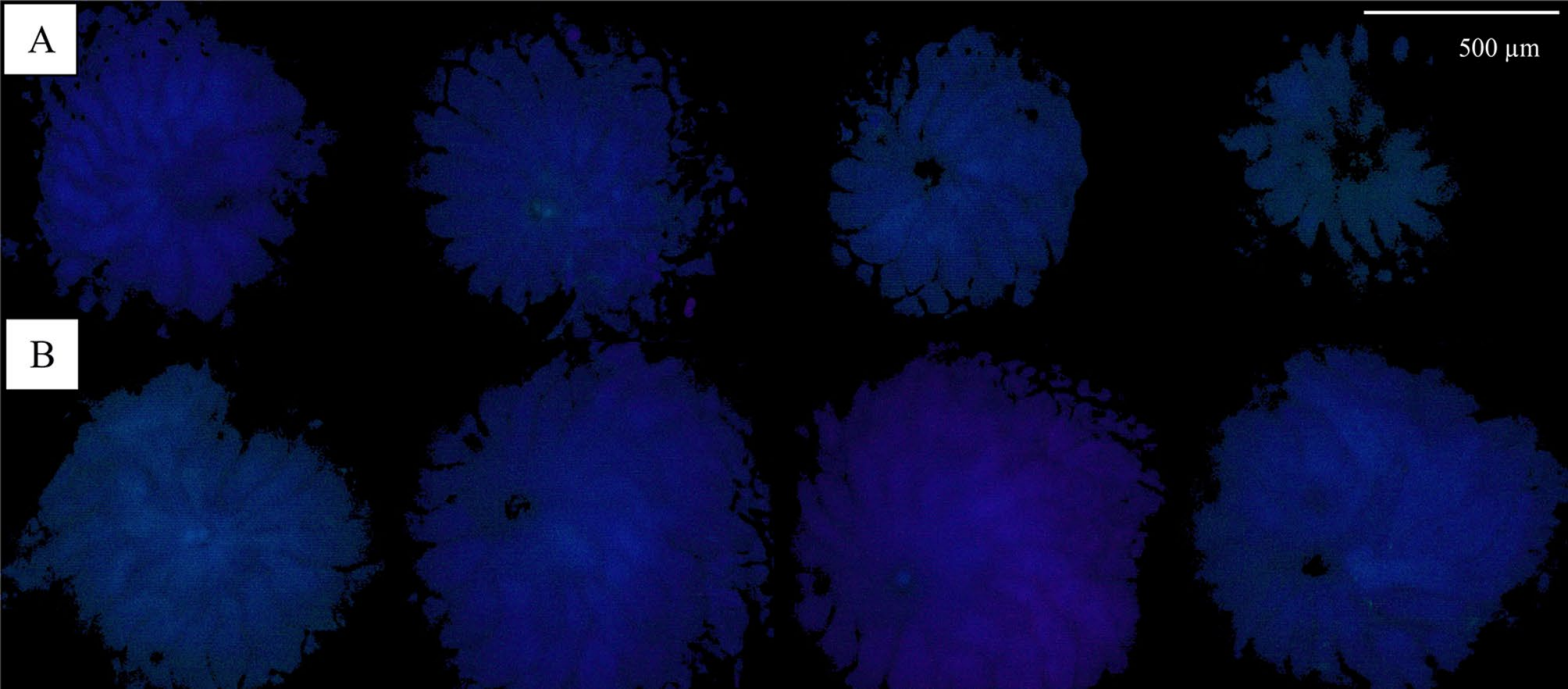
Evaluated PAM-images recorded the change of photosynthetic active areas with time (left to right: 0, 1, 3, and 7 days). The first images (**A**) are an example for a sample which contain sunscreen (in this case sunscreen D50, see Table 2). The second one (**B**) shows the variation of the control.

**Figure 2:**
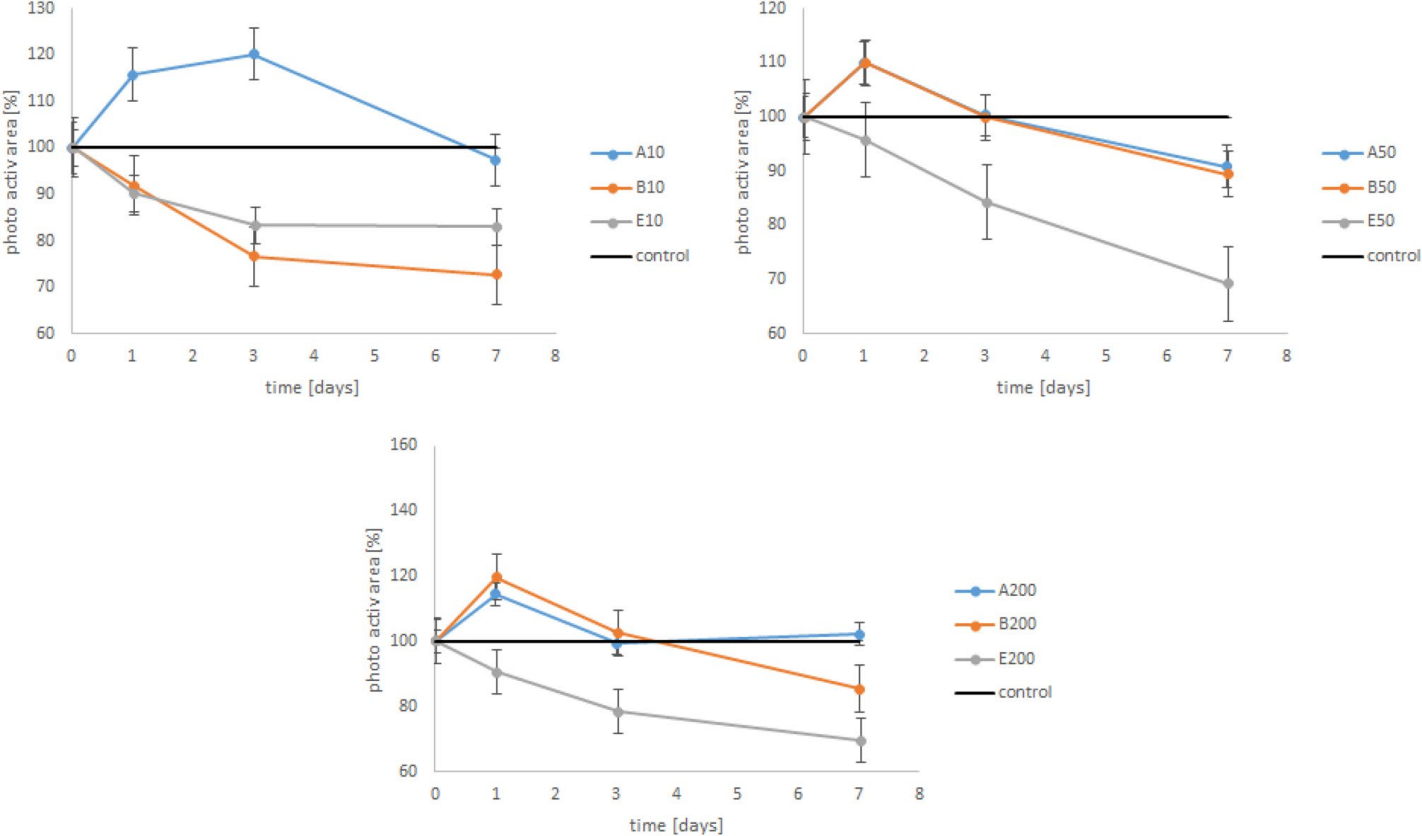
Mean photosynthetic area of the tested foraminifera (six replicates, error bars indicate standard error) with conventional sunscreens in relation to the control (black line, where the mean of six replicates relates to 100%). 10, 50 and 200 stand for 10, 50, and 200 mg/L sunscreen, A, B and E are described in Table 1.

The photosynthetic area changed significantly with time (p < 0.001, Df = 6) (Fig. 1), applied conventional sunscreen (p = 0.025, Df = 2) and concentration (p = 0.010, Df = 2) (Fig. 2). Ensulizole had a negative impact on the photosynthetic area even at the lowest concentration E10 (Fig. 2). For the other experiments (except B10) a slight stimulation (increasing of photosynthetically active area) of foraminifera was recognized within the first day (A10 even to day 5). After that time, the sunscreen lines showed also a negative trend in comparison to the control. The experiments with pure Ensulizole showed highly significant time-dependency (p < 0.001), but no significant concentration effect was found (p = 0.086).

### The “eco-friendly” sunscreens

**Figure 3:**
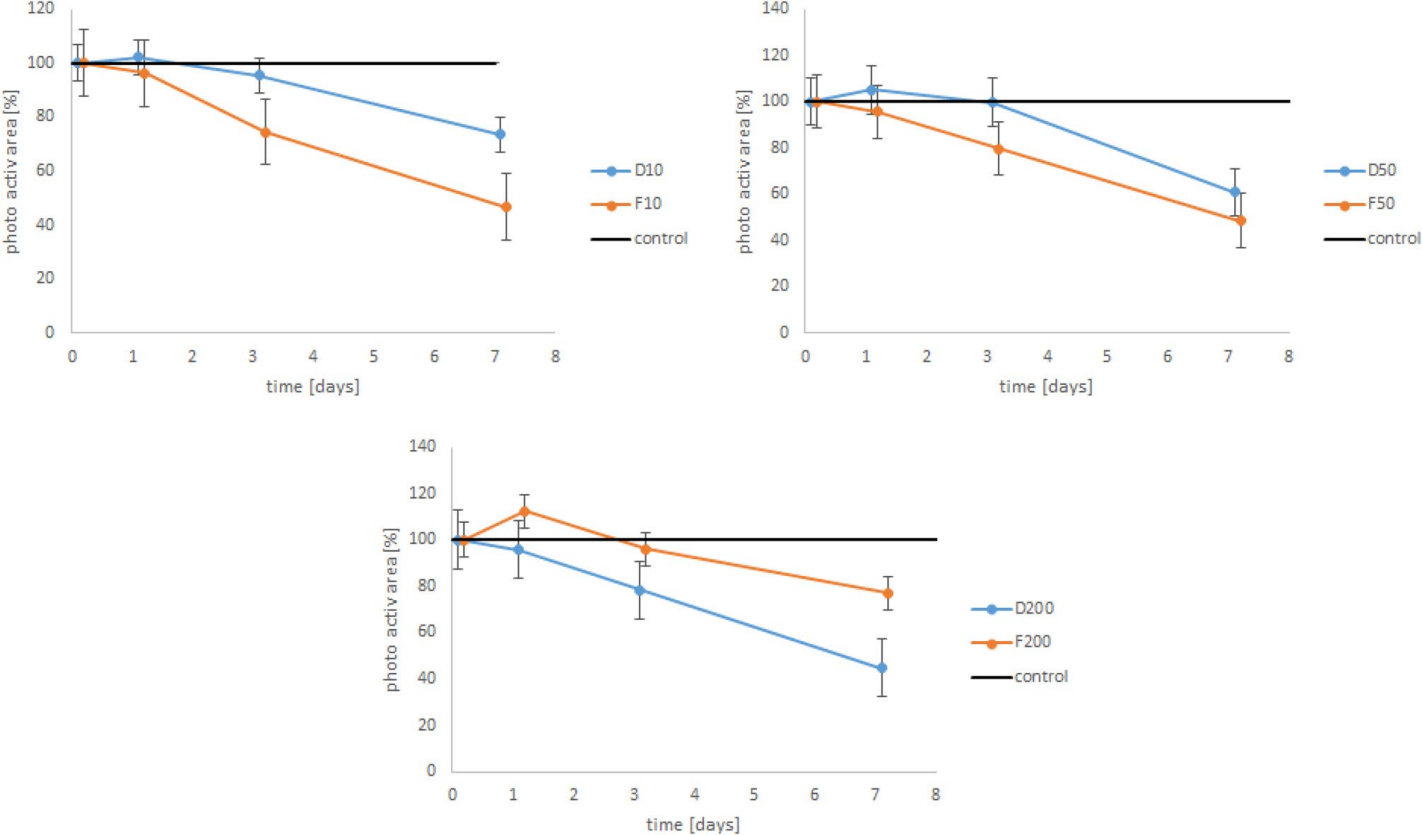
Mean photosynthetic area of the testes foraminifera (six replicates, error bars indicate standard error) with „eco-friendly “ sunscreen compared to the control (black line, where the mean of six replicates relates to 100%). 10, 50 and 200 stand for 10, 50, and 200 mg/L sunscreen, D and F are described in Table 1.

During the first days a slight increase of the photosynthetic area was observed, followed by a strong decline (Fig. 3). No significant difference (p = 0.541, Df = 1) was found between the two “eco-friendly” sunscreens. Within the tested sunscreens a highly significant difference in time (p > 0.001) was observed. The photosynthetic area depends also on the concentration of sunscreen (A: p = 0.002, Df = 2; B: p > 0.001, Df = 2).

### Comparison conventional and “eco-friendly” sunscreens

**Figure 4:**
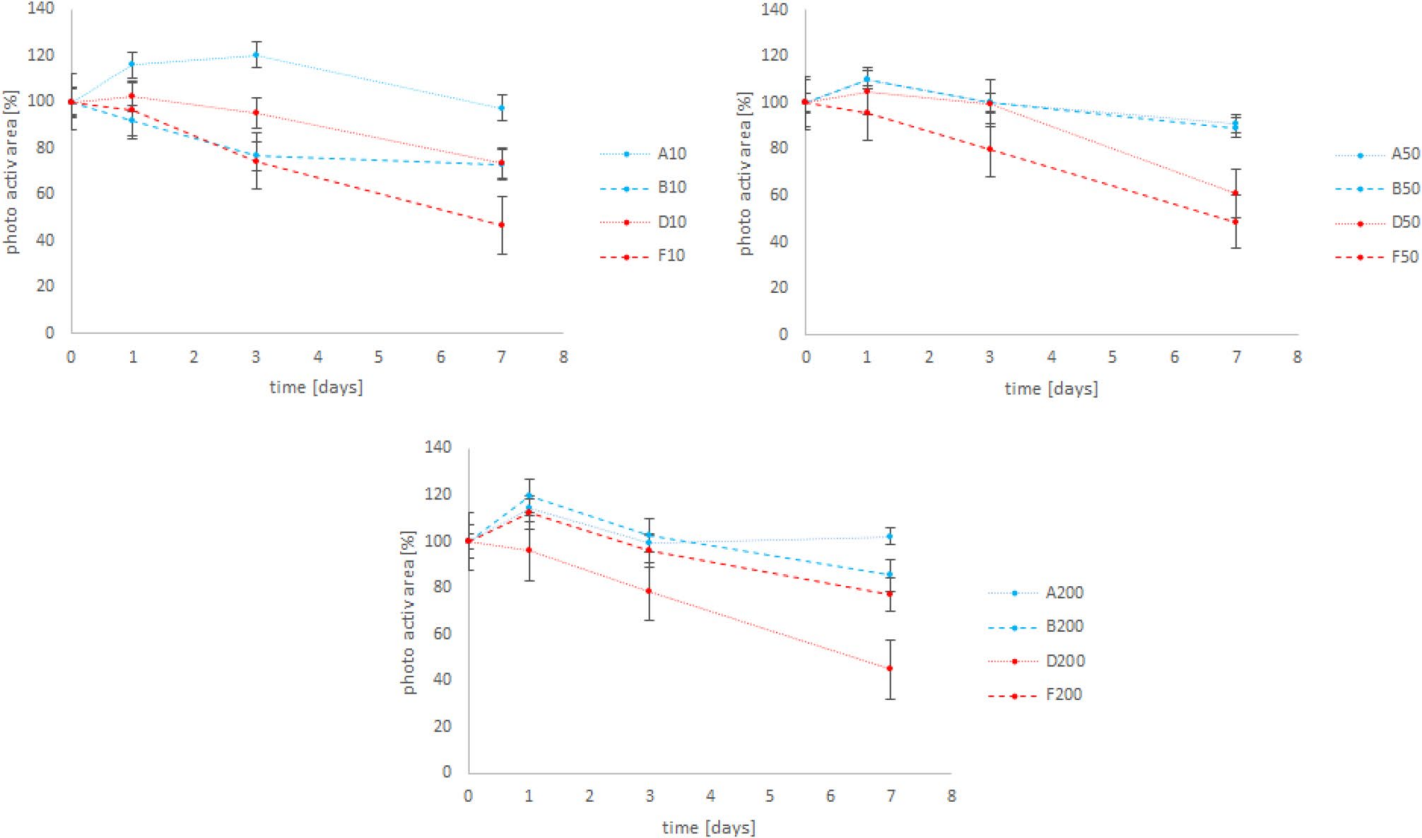
Mean photosynthetic area of the tested foraminifera (six replicates, error bars indicate standard error). A and B are the conventional and D and F the “eco-friendly” sunscreens (see Table 1 for more detail). The values are in percent to the control (control values are 100%).

Figure 4 shows combinations of Figs. 2 and 3 and allows a comparison of conventional and “eco-friendly” sunscreens at a glance. Between the conventional and the “eco-friendly” sunscreen a significant difference of the photosynthetically area with time can be observed (p > 0.001, Df = 1). With longer duration of the experiments and higher concentrations of sunscreens, this difference becomes clearer.

## Discussion

### The effect of conventional sunscreens on foraminiferal symbiont activity

Increased concentrations of sunscreen have been reported in 95% of wastewater effluents, in 86% of surface waters and in the tissues of aquatic biota around the world^24^. It could be estimated, that about 25% of the total used sunscreen is washed directly into the coastal areas during swimming, washing or showering^25^. But not all substances are toxic for the environment in the same way. Brausch and Rand^26^ found out, that in such sunscreen-wastewater treatments plants were enriched with four substances. These substances are 2-ethyl-hexyl-4-trimethoxycinnamate (EHMC), 4-methylbenzylidene camphor (4MBC), benzophenone-3 (BP3) and octocrylene^26,27^. The conventional sunscreens A and B did not contain any of these four substances, but they contained Ensulizole. Godejohann et al.^28^ found that Ensulizole gets incorporated into plant cells. We observed a negative effect of pure Enulizole for the photosynthetic symbionts of the tested foraminifera at each concentration. However, no significant concentration effect by the pure Ensulizole treatments was observed. That leads to the assumption, that even the lowest concentration (10 mg L^−1^) is already strongly inhibiting the activity of the algae. The concentrations of Ensulizole in the sunscreens are lower than in the lowest treatment of pure Ensulizole. The conventional sunscreens themselves showed just a slightly negative effect on the foraminiferal symbionts, which can be explained by these lower concentrations. In the first days, even a stimulation of the activity was recognized. As this pattern was found in every setup (expect B10), we assume that water-soluble substances of the sunscreens boosted foraminiferal activity, which was not further tested in this study. After that stimulation, the foraminiferal activity stayed constant or decreased, regarding the reduced amount of photosynthetic area, which indicates, that foraminifera were stressed in that time. During the exposure to Ensulizole, it looks like foraminifera are even able to recover after the E10 and E50 setup, but not in the E200 setup. This implies, that a concentration of 200 mg L^−1^ Ensulizole is definitively toxic for the symbionts of *H. depressa*, causing the death of the foraminifera.

### The effect of “eco-friendly” sunscreens on foraminiferal symbiont activity

As mentioned in the introduction, LBFs are important organisms in coral reefs and play a key role in the carbonate production there. Further, with the presence of LBFs it is possible to define the health of a reef by calculating the FORAM (Foraminifera in Reef Assessment and Monitoring) Index following Hallock et al.^29^. The influence of sunscreen pollution on reefs, based on the FORAM Index cannot be calculated yet, because in this study only the effect of one species (*H. depressa*) and not the whole foraminiferal community was investigated. At the moment, an assumption can be made that an increase of sunscreen in the seawater will lead to a decrease of the amount of living LBFs and therefore the FORAM Index will drop down, but of course further studies are necessary to clarify this aspect.

Most of “eco-friendly” contain no Ensulizole but metal compounds such as titanium dioxide or zinc oxide (see ingredients of the products). In the last decade the usage of titanium dioxide nanoparticles increased strongly^30^. Nanoparticles are able to aggregate and become a possible risk for the organisms in the environment (e.g.^31^). This aggregation is affected by several parameters like pH or ionic strength (e.g.^30^). Sharma^30^ found a high mortality (60%) of *Daphnia magna* at titanium dioxide concentration of 20 g L^−1^. Actually, there are no studies dealing with the interaction of TiO_2_–nanoparticles and foraminifera. Still, nanoparticles may even interact with DNA, lipids, proteins and tissues of organisms (e.g.^32^). Titanium dioxide however occurs also in nature, e.g., in metamorphic rocks, including the minerals ilmenite (FeTiO_3_) and rutile (TiO_2_), which can also be found in the beach sediment^33^. However, titanium dioxide nanoparticles concentrations from natural sources are in a range of just a few µg L^−1^^34^.

The second potentially toxic metal is zinc oxide, which was also an ingredient of our tested sunscreens. Zinc oxide has a toxic effect on coastal living diatoms^35^. Beside its UV-absorption properties^36^, zinc oxide nanoparticles are commonly used as antimicrobial agents^37^. This points at the toxic potential impact on organisms. Not only zinc oxide nanoparticles have a negative impact on foraminifera, even dissolved zinc from the water-soluble zinc sulfide reduces their metabolism (Lintner et al. 2021).

Our tested sunscreen D contained titanium dioxide and F contained both titanium dioxide and zinc oxide. We observed, that the “eco-friendly” sunscreens had a more negative impact on the foraminifera, than the conventional ones (see Figs. 2 and 3), which indicated that the presence of nanoparticles and/or heavy metals in the environment can lead to a reduction of the metabolic activity of *H. depressa*. This negative effect was also observed on other microorganisms like for example diatoms^35^ which are the most common symbionts in LBFs (e.g.,^12,38^). Considering, that sunscreen F contained zinc oxide and sunscreen D not, there was no great difference in the pattern of the reduction of the metabolisms. This indicates, that the potential toxic substance in this sunscreen is titanium dioxide rather than zinc oxide.

## Conclusion

Not only environmental parameters like salinity or temperature are affecting the health of foraminifera, but also substances of anthropogenic origin. We found that sunscreens reduce the metabolism of foraminifera, based on the decreasing amount of photosynthetic active symbionts, which are obligatory for large benthic foraminifera. From the results obtained, we conclude that certain substances like Ensulizole and heavy metal nanoparticles lead to a substantial decrease of the photoactive area in *Heterostegina depressa*. Due to the fact, that this area indicates obligatory symbionts, the decrease of the photoactive area will finally result in an increased mortality of foraminifera. Not all tested sunscreens had the same negative impact on foraminifera. The metal nanoparticles (presumably titanium dioxide) in “eco-friendly” sunscreens decreased the photoactive area of *H. depressa* strongly with time. Also, the organic substance Ensulizole, which can be found in conventional sunscreens, had a strong negative impact on the health of foraminifera, especially when the culture is highly enriched (> 50 mg/L) with this substance. In conventional sunscreens the Ensulizole concentration is lower than 50 mg/L and therefore the negative impact on the foraminifera is much lower with time in contrast to the pure Ensulizole incubation experiments. However, it seems that the “eco-friendly” sunscreens have a higher negative impact on the LBFs and their symbionts than the conventional ones, which leads to the assumption that metal nanoparticles are more toxic for these kinds of foraminifera than Ensulizole. Since large benthic foraminifera like *H. depressa* are essential components in the marine ecosystem and important for carbonate production, it is of particular interest to examine them more closely. Based on the results of this study, conclusions can be drawn about the health of marine shallow water communities due to the lack or presence of such kind of foraminifera. Further, our observations are essential for water quality monitoring, since foraminifera are often used as proxies to define certain physical parameters in marine habitats.
